# Immune response to arbovirus infection in obesity

**DOI:** 10.3389/fimmu.2022.968582

**Published:** 2022-11-18

**Authors:** Muddassar Hameed, Elizabeth Geerling, Amelia K. Pinto, Iqra Miraj, James Weger-Lucarelli

**Affiliations:** ^1^ Department of Biomedical Sciences and Pathobiology, VA-MD Regional College of Veterinary Medicine, Virginia Tech, Blacksburg, VA, United States; ^2^ Department of Molecular Microbiology and Immunology, Saint Louis University, St. Louis, MO, United States; ^3^ College of Biosystems Engineering and Food Science, Zhejiang University, Hangzhou, China

**Keywords:** obesity, arboviruses, adipocytes, cytokines, interferons

## Abstract

Obesity is a global health problem that affects 650 million people worldwide and leads to diverse changes in host immunity. Individuals with obesity experience an increase in the size and the number of adipocytes, which function as an endocrine organ and release various adipocytokines such as leptin and adiponectin that exert wide ranging effects on other cells. In individuals with obesity, macrophages account for up to 40% of adipose tissue (AT) cells, three times more than in adipose tissue (10%) of healthy weight individuals and secrete several cytokines and chemokines such as interleukin (IL)-1β, chemokine C-C ligand (CCL)-2, IL-6, CCL5, and tumor necrosis factor (TNF)-α, leading to the development of inflammation. Overall, obesity-derived cytokines strongly affect immune responses and make patients with obesity more prone to severe symptoms than patients with a healthy weight. Several epidemiological studies reported a strong association between obesity and severe arthropod-borne virus (arbovirus) infections such as dengue virus (DENV), chikungunya virus (CHIKV), West Nile virus (WNV), and Sindbis virus (SINV). Recently, experimental investigations found that DENV, WNV, CHIKV and Mayaro virus (MAYV) infections cause worsened disease outcomes in infected diet induced obese (DIO) mice groups compared to infected healthy-weight animals. The mechanisms leading to higher susceptibility to severe infections in individuals with obesity remain unknown, though a better understanding of the causes will help scientists and clinicians develop host directed therapies to treat severe disease. In this review article, we summarize the effects of obesity on the host immune response in the context of arboviral infections. We have outlined that obesity makes the host more susceptible to infectious agents, likely by disrupting the functions of innate and adaptive immune cells. We have also discussed the immune response of DIO mouse models against some important arboviruses such as CHIKV, MAYV, DENV, and WNV. We can speculate that obesity-induced disruption of innate and adaptive immune cell function in arboviral infections ultimately affects the course of arboviral disease. Therefore, further studies are needed to explore the cellular and molecular aspects of immunity that are compromised in obesity during arboviral infections or vaccination, which will be helpful in developing specific therapeutic/prophylactic interventions to prevent immunopathology and disease progression in individuals with obesity.

## Introduction

Obesity is defined as an excessive accumulation of body mass or an increased mass of adipose tissue beyond the body’s requirement (1). According to a 2016 survey by WHO, 1.9 billion adults are overweight and the number of people with obesity has tripled since 1975 (2). Importantly, obesity is spreading rapidly worldwide due to numerous obesity-promoting factors such as physical inactivity, high caloric foods and drinks, and changing lifestyle habits due to internet use, smartphones, video games, etc. (3). This high prevalence increases the incidence of metabolic and cardiovascular diseases common in people with obesity such as type 2 diabetes mellitus, osteoarthritis, and hypertension (1, 4). It is thought that excessive adipose tissue contributes to increased incidence of associated diseases by promoting a chronic inflammatory state (5–7).

In addition to metabolic impacts, obesity has been identified as an independent risk factor for severe viral diseases such as influenza and coronavirus disease 2019 (COVID-19) (8–12). Epidemiologic data from the COVID-19 pandemic in the United States showed that individuals with higher body mass index (BMI ≥30-35 kg/m^2^) were more likely to be admitted to the intensive care unit compared with individuals with a BMI of <30 (13, 14). Several studies from different part of world such as Singapore, France, England and China reports that the COVID-19 produces severe signs and symptoms in individuals with obesity compared to lean people and was also associated with a higher risk of COVID-19-associated death (15–18). A similar pattern was observed during the 2009 H1N1 influenza pandemic, in which obesity was first reported as an important comorbidity for increased disease severity and deaths (12, 19). Obese people with history of respiratory diseases becomes more susceptible to influenza and COVID-19, which is not same for arboviruses. The role of obesity in susceptibility to arbovirus diseases should take a note of caution.

There are very few data on the impact of obesity on arbovirus infections. Arboviruses cause significant disease each year; dengue virus, for example, is endemic in 129 countries and causes 390 million infections per year (20, 21), ZIKV caused more than 220,000 confirmed cases in 52 countries or territories in the America (22, 23), and CHIKV and WNV have spread to a number of countries, resulting in millions of cases (24–30). Several seroprevalence studies have found a strong association between obesity and previous arbovirus infections. In Madagascar, seroprevalence of antibodies to DENV, CHIKV, and Rift Valley fever virus (RVFV) was studied. This showed that CHIKV infection was significantly associated with higher body weight (31). Similar data were reported from La Réunion and India for CHIKV infections, where individuals who were overweight or obese had a higher risk of disease compared to the healthy population (32, 33). In addition, obesity has also been associated with higher seropositivity for DENV in Thailand (34), Sindbis virus (SINV) in Sweden (35), and Toscana virus (family *Phenuiviridae*) and Sicilian phlebovirus (family *Phenuiviridae*) in Italy (36). It is reported that obesity contributes to increase disease severity by promoting a chronic inflammatory state (5–7). It has also been reported that arboviral infection (DENV) is higher in obese people because Adenosine Monophosphate (AMP)-Activated Protein Kinase (AMPK) is down regulated in obesity which is a major regulator of cellular energy homeostasis (37, 38). Arboviruses downregulates AMPK activity to prevent lipid metabolism and increase lipid quantities available to form the lipid envelope during viral replication (38, 39). Obese individuals already have a low AMPK activity, which is further downregulated by viral infection in order boost ER cholesterol levels thus facilitating viral replication that could lead to more severe disease. In addition, the development of severe infections may be due to impaired CD8+T and natural killer (NK) cell activity in obese hosts (40–42). Efforts are continued for the development of host directed effective therapeutics such as the use of metformin in overweight and obese young dengue patients to monitor its effects on viral replication, endothelial dysfunction, and host immune responses (43). Recently, several laboratory studies report that DENV, WNV, and CHIKV (as well as other related alphaviruses) infections cause more severe symptoms in infected obese mice compared with infected healthy weight animals (44–46). In this review article, we describe the immunological impairment against arboviral infections in patients with obesity and discuss the implications on disease severity.

### The effect of obesity on immunity

In obesity, more adipose tissue accumulates in the body. There are two main types of adipose tissue, white adipose tissue (WAT), which plays an important role in energy storage, and brown adipose tissue (BAT), which has an important function in thermogenesis. Adipocytes are the major cell types of adipose tissue and are further subdivided based on their microscopic appearance (47, 48). In WAT, a unilocularly arranged lipid vacuole predominates, whereas in BAT, multilocular lipid vacuoles are present and intermediate cell forms are referred to as beige adipocytes (49). In addition to adipocytes and pre-adipocytes, fibroblasts, endothelial cells, leukocytes, and macrophages are also part of adipose tissue (50). The number of macrophages correlates positively with body mass, adipocyte size, and expression of pro-inflammatory cytokines (51, 52).

**Figure 1:**
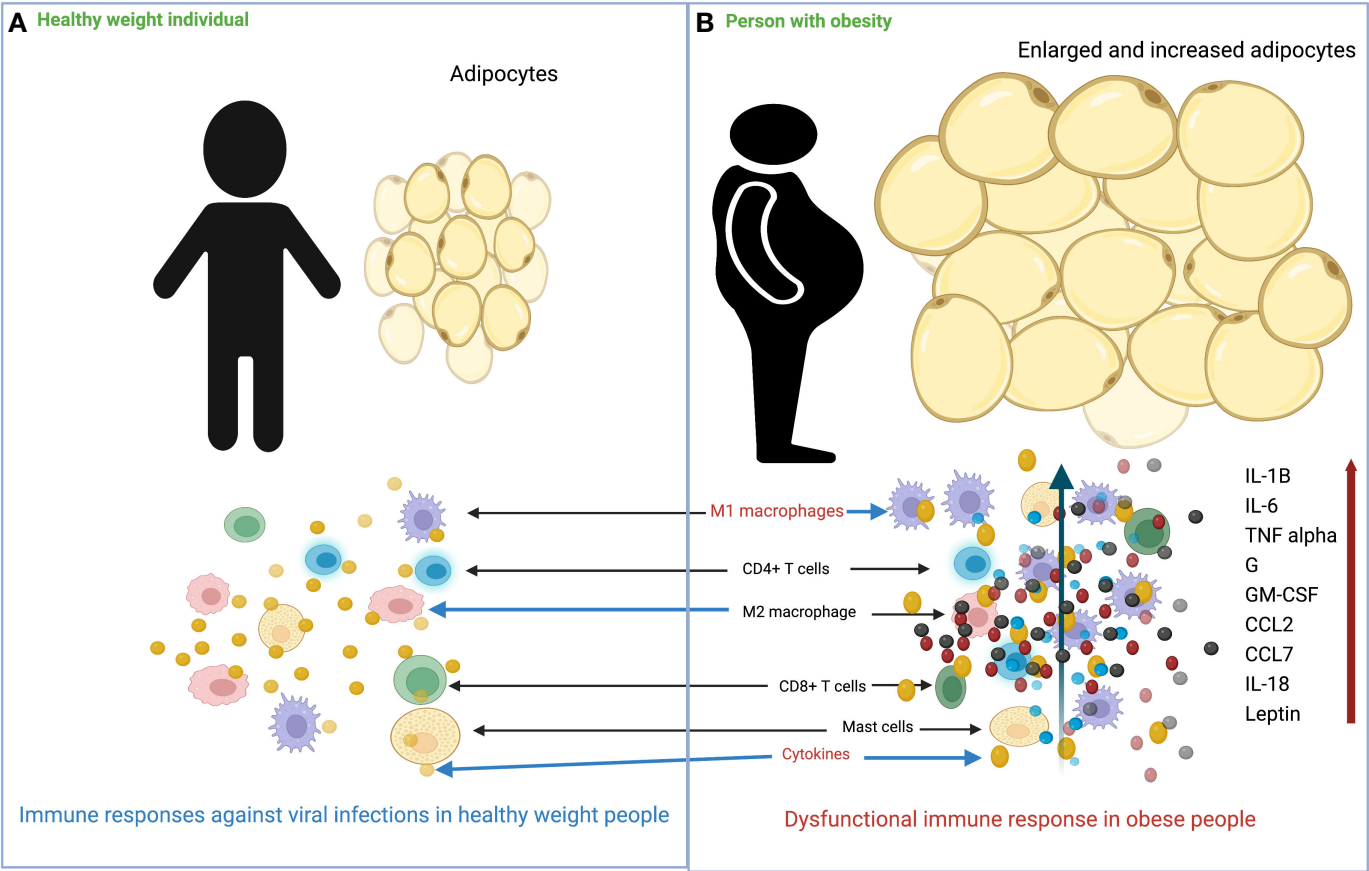
Overview of the differences in immune response to viral infection in healthy weight and obese individuals. **(A)** In healthy weight individuals, adipocytes are smaller and less numerous, and the immune system responds normally to viral infection. **(B)** In individuals with obesity, adipocytes increase in size and number, and there is massive infiltration of M1 macrophages into adipose tissue. Following viral infection, the immune system responds with impaired production of various cytokines/chemokines.

**Figure 2:**
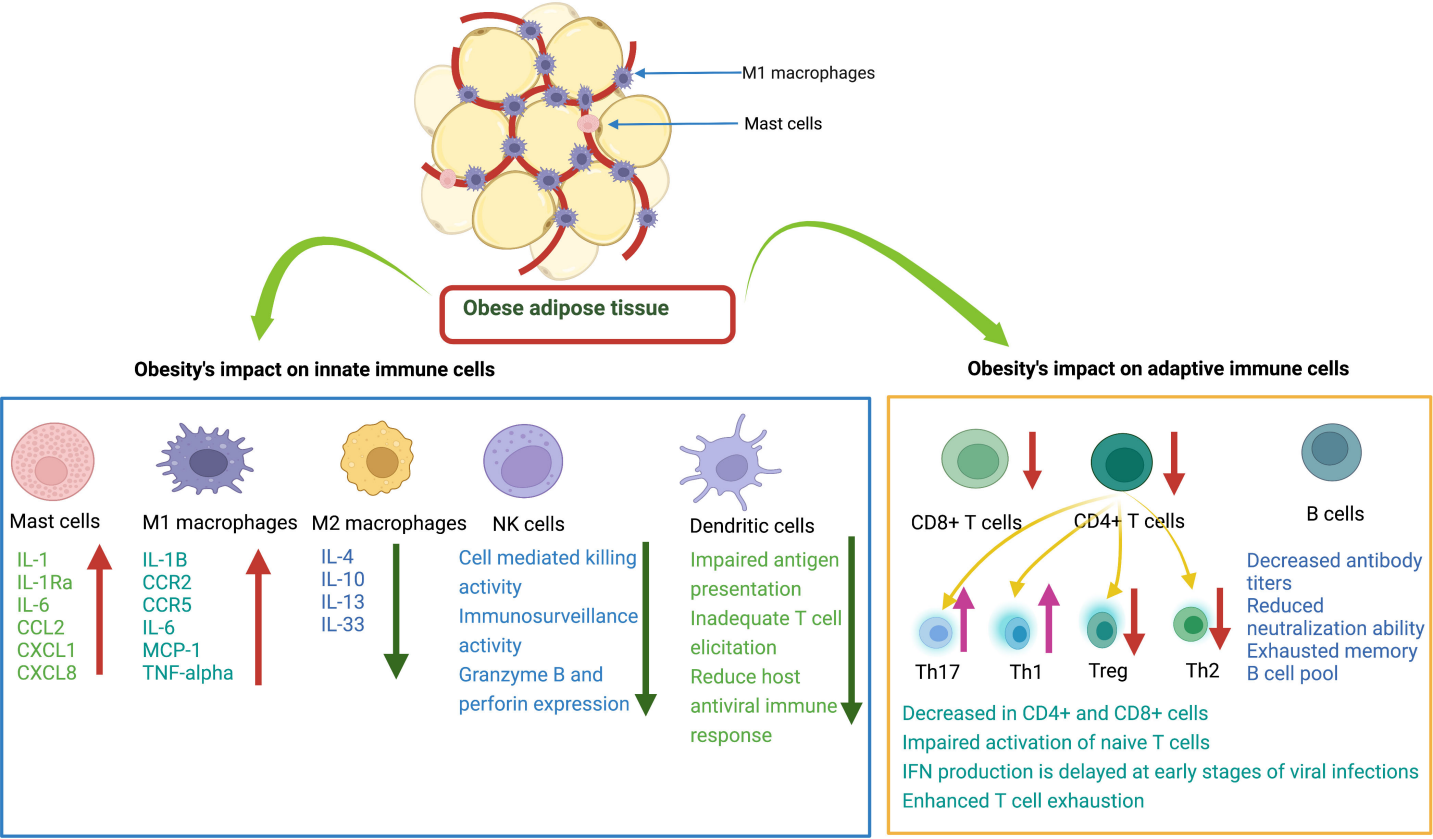
Effects of obesity on innate and adaptive immune cells. Mast cells (MCs) release various cytokines and chemokines that promote inflammation. M1 macrophages release proinflammatory mediators. M2 macrophages release anti-inflammatory cytokines. Natural killer cells decrease immune surveillance activity. Dendritic cells have impaired ability to present antigens to other immune cells. CD8+ T cells have decreased cytotoxic activity to kill infected cells. CD4+ T cells: there is a decrease in Treg and Th2 cells and an increase in Th17 and Th1 cells. B cells have decreased antibody production with reduced neutralizing ability.

In people with obesity, an increase in adipose tissue is characterized by an increase in size (hypertrophy) and the number (hyperplasia) of adipocytes. Adipocyte hypertrophy is accompanied by inadequate vascularization, which creates hypoxic conditions in adipose tissue, induces apoptosis or necrosis, and increases secretion of inflammatory cytokines, chemokines, and adipokines, leading to severe infiltration of immune cells, as shown in **Figure 1** (53–58). In parallel with this increase in size of adipocytes, adipose tissue undergoes a remodeling phase with overproduction of extracellular matrix (ECM) and increased infiltration of immune cells (59, 60). The interaction between adipocytes and macrophages and the metabolic inflammation triggered by macrophages play an important role in the remodeling process (61–64). Scientists have proposed that the most critical step in obesity-related infections is the initiation of macrophage migration into adipose tissue, which could be triggered by adipocyte death, hypoxic conditions, chemotactic regulation, and fatty acid flux, eventually leading to a state of low-grade chronic inflammation (59, 63, 65). Previous data also suggest the distinction between pathological and healthy adipose tissue development. In pathological expansion, the existing adipocytes increase rapidly, resulting in hypoxia due to decreased blood vessel formation, deposition of ECM, and increased infiltration of more pro-inflammatory M1 type macrophages, whereas, more anti-inflammatory M2 macrophages predominate in normal body weight AT and adequate oxygen supply and normal levels of immune cells and cytokines are maintained (66–68) (**Figure 2**).

Adipose tissue (AT) also has important endocrine functions and secretes different immune mediators that play a role in immune cell infiltration and disease following infection. These mediators include adipocytokines or adipokines such as leptin, adiponectin, resistin, visfatin and other important immunological factors such as tumor necrosis factor (TNFα), IL-1, IL-6, plasminogen activator inhibitor type I (PAI-I), CCL2, and different complement factors (50, 69–72). The increased level of adipokines such as leptin activates intracellular genes through different signaling pathways such as JAK-STAT, MAPK, PI3K, and AMPK, leading to the production of various intracellular inflammatory cytokines (73–76). In people of a healthy weight, regulatory anti-inflammatory cells and cytokines such as Treg, Th2 cells and IL-4, IL-5, IL-10, IL-14, IL-33, are maintained to control excessive inflammation (77–79). However, in individuals with obesity, this regulatory, anti-inflammatory state of the immune system transforms into an inflammatory state by secreting various pro-inflammatory cytokines, including IL-1β, IL-6, IL-8, TNFα, and CCL2 (65, 77, 80, 81). These pro-inflammatory cytokines contributes to obesity-induced chronic low-grade inflammation.

Furthermore, obesity is also associated with the senescence of immune cells, which promote the release of pro-inflammatory cytokines (82, 83). Moreover, the role of microRNAs (miRNAs) in obesity has been explored and unmasked as an important biomarker for obesity (84, 85). MiRNAs affect the expression and regulation of many protein-coding genes involved in the regulation of inflammatory processes (86–88). MiRNA-146a, for example, interferes with the activation of nuclear factor kappa B (NF-κB) induced by TNFα and Toll-like receptor ligands (TLR), while miRNA-155 promotes the activation of LPS/TNF pathways (88–90). In conclusion, the excess adipose tissue in obesity activates intracellular signaling pathways, leading to the production of pro-inflammatory cytokines, and promotes the infiltration of more M1 phenotype pro-inflammatory macrophages. Overall, this section highlights the cellular and molecular differences in immune and inflammatory mediators between individuals with obesity or of healthy weight.

### The influence of obesity on the generation of immune responses after viral infection

After viral infection, the host activates innate and adaptive immune cells to generate an antiviral immune response against the invading pathogen. In people with obesity, excessive fat deposition in immune tissues such as the spleen, thymus, lymph nodes, and bone marrow alters the cellular environment and disrupts the integrity of tissue, impairing proper development and maturation, diversity, phenotype, and activity of immune cells (91–93). This abnormal development of immune cells leads to impaired interferon and cytokine production, which impairs host antiviral immunity and increases the risk of severe viral disease in patients with obesity (91, 94).

Previously, O’Shea et al. investigated the characteristics of dendritic cells (DCs) in obesity and found that the number of circulating DCs decreased significantly in individuals with obesity and their abnormal function was characterized by decreased expression of CD83 after TLR stimulation compared to the control group (95). CD83 plays a critical role in triggering T cell responses, and its decreased expression leads to a weakened host antiviral immune response and increased severity of viral infections in patients with obesity (95–97). Experimental data show that DCs in DIO mice have a blunted ability to trigger the expansion of naïve T cells due to higher levels of cytokines and chemokines such as IL-1α, IL-17, and TNFα (98, 99). During influenza virus infection, DCs from patients with obesity showed impaired antigen presentation and insufficient competence in directing antiviral orchestration of T cells (100, 101). This abnormal state was associated with increased pro-inflammatory cytokine levels in the lungs of DIO mice, particularly the IL-6 associated proinflammatory state that impairs the number and frequency of CD4+ and CD8+ T cells in the lungs. Furthermore, obesity has also been reported to impair the migration of DCs to the lymph nodes (102).

Studies have shown that humans or animals with obesity have functionally impaired NK cells, which increase the risk of cancer and viral infections (103–107). With the development of obesity, specific lipid uptake receptors expression is increased in NK cells which results in increase uptake of free fatty acids and activated NK cells fail to activate mTOR and glycolytic metabolism resulting in decrease IFN- γ production which is reviewed in detail previously (108). Veil and colleagues experiment data reveals the increased expression of activation markers such as CD69 on NK cells from obese patients which result in altered degranulation and reduced production of IFN- γ (107). Nave et al., observed that leptin treatment stimulated NK activity four times higher in lean than obese animals (41). The activation of post receptor signaling components (Janus kinase-2p, protein kinase B pT308, AMPalphapT172) was reduced after an *in vivo* leptin challenge in obese animals (41). Recently, it is reported that impaired NK cell function, and polymorphisms in NK cell cytolytic function genes are associated with hyperinflammation which enhances dengue severity (109). Collectively, these findings provide evidence obesity induced disturbance in NK cells function can lead to severe arboviruses diseases in obese patients which remains unknown.

There are currently limited data on the response of interferons/cytokines to arbovirus infection in obese humans/animals. It has been reported that humans with obesity do not elicit a robust type I IFN response in viral infections such as H1N1 influenza virus infection (110–112). Cabanillas et al. demonstrated that obese mice infected with H1N1 virus had significantly lower levels of IFN-α and IFN-β compared with the control group, and viral load and mortality were also higher (111). Obese H1N1 patients were also found to have decreased IFN-α production, which could be related to leptin levels, leading to dysregulated development of immune cells and their shift toward inflammatory phenotypes (42, 113). Leptin is chronically produced by AT in individuals with obesity and interferes with IFN signaling by increasing Suppressor of Cytokine Signaling 3 (SOCS3) (114). SOCS3 negatively regulates the JAK-STAT signaling pathway and limits IFN production by downregulating interferon stimulated gene (ISG) transcription in individuals with obesity (113, 115). Costanzo et al. reported that IFN-γ production was decreased in individuals with obesity infected with influenza A virus, which was due to dysfunctional γδT cells (116). In addition to the abnormal number of γδT cells during influenza virus infection in individuals with obesity, the surviving cells also become unresponsive to TLR ligands, further contributing to the IFN deficiency (116). This IFN deficiency could block the IFN signal transduction cascade. A comparative study of cytokine production after influenza virus infection revealed that the production of cytokines such as IL-6, TNF-α, IL-1β, and CCL-2 was delayed and decreased in the early stages of infection in obese subjects (103, 111, 117). This delayed immune response leads to low-grade chronic inflammation (112). However, in the later stages of infection, excessive cytokine secretion in obese subjects leads to cytokine storm (103, 117, 118). Thus, obesity-induced chronic inflammation and deregulated immune response lead to impaired clearance of viral particles in the early stage of infection.

The release of inflammatory mediators from adipose tissue is obesity such as leptin, adiponectin, resistin, visfatin and other important immunological factors such as tumor necrosis factor (TNFα), IL-1, IL-6, plasminogen activator inhibitor type I (PAI-I), CCL2, and different complement factors produce obesity-induced chronic inflammation (50, 69–72). This chronic inflammation leads to the T cells exhaustion through different ways. First, T cells from humans and animals with obesity exhibit decreased proliferative capacity and increased exhaustion, as evidenced by downregulated Ki67 and upregulated PD-1 (119). The higher leptin levels in obesity lead to the upregulation of phosphorylated STAT3, which induces PD-1 expression in T cells. This is also demonstrated by decreased IFN-γ and TNFα production in stimulated polyclonal T cells from individuals with obesity (119). Second, increase in glucose, FFAs, phospholipids, cholesterol, and other metabolites in individuals with obesity alters the metabolism of T cells, leading to impaired activation and decreased activity (118, 120, 121). Moreover, IFN production is delayed in the early stages of viral infections in obesity, and this delayed IFN production inhibits T cell proliferation, blocks their efflux from lymphoid organs, and leads to T cell exhaustion (122–124). The disruption of the antiviral immune response could increase cell apoptosis and impair normal T cell activation and proliferation during viral infections. Several studies have shown that patients with obesity infected with influenza or COVID-19 who become severely ill lack an effective antiviral T cell response (125–127).

The immune system of patients with obesity is also inherently weakened due to immune senescence (82, 83). The development of obesity induces oxidative stress and inflammation, which shortens telomere length and leads to cellular aging (128, 129). In adiposity, leptin levels increase in serum which also cause the telomere shortening (130, 131). Epigenetic studies reveal that obesity induces a widespread gene expression and methylation changes in multiple tissues of body including blood leukocyte DNA, which can cause immune dysfunction (132–134). It has been reported that senescent cells expressing SA β-gal activity and p53 levels increase in obese animals (135, 136). It was seen that depletion of senescent cells from obese animals can ameliorate pathology (137, 138). In addition to the association between obesity and disease progression due to a reduction in T cells, obesity also promotes thymic degeneration and T cell senescence, which is seen in elderly individuals with obesity and even in children with obesity (139–141). There is epigenetic evidence of hypermethylation of T lymphocyte DNA in humans and animals that exhibit obesity-related T cell senescence (142, 143). Regulatory T cells are found to be important regulatory cells in AT that provide anti-inflammatory signals (99, 144). In an experimental study, Feuerer et al. found that when most Treg cells were removed from AT, pro-inflammatory transcripts were overexpressed compared to the experimental group, suggesting the key anti-inflammatory role of Treg cells (78). γδT cells also produce growth factors, induce maturation of DCs, recruit macrophages, and interact with Treg cells (145). In patients with obesity, the number of γδT cells is decreased, which could lead to an impaired antiviral response and worsening of disease pathology due to their sensitivity to inflammation (116). Impaired T-cell response in obesity caused by a combination of host and viral factors predisposes individuals with obesity to failure of viral control and development of severe disease (**Figure 2**).

B cells play a critical role in limiting viral replication and dissemination through antibody production. Non-neutralizing antibodies perform essential tasks, often through their constant (Fc) region, in ways like interacting with complement proteins to enhance opsonization or through Fc receptor interactions which mediate antibody-dependent cellular cytotoxicity. Similarly, neutralizing antibodies also serve essential functions in the immune response to pathogens by blocking viral entry into host cells. Previous experiments have shown that in individuals with obesity exposed to H1N1 influenza virus, the titer of virus specific antibodies is reduced, and antibodies primed in the obese state have a weakened neutralization capacity compared to those primed in healthy weight individuals (146, 147). This could be due to several factors, such as greater inflammation, DNA hypermethylation of B cells, and abnormal leptin levels that differentially regulate B cell development, maturation, and activity (142, 143, 148). It has been described that the peripheral B cell pool of individuals with obesity contains a higher proportion of pro-inflammatory late/exhausted memory B subsets and a lower proportion of anti-inflammatory transitional B cells (148, 149). Moreover, functional defects of B cells in individuals with obesity contribute to triggering an acute inflammatory state through the production of pro-inflammatory mediators. It is plausible that obesity leads to severe arbovirus infections by altering the number and function of B cells and the potential interaction with other lymphocytes (follicular T helper cells) by producing a hyperinflammation cascade and an imbalance of adipokines. Overall, the development of obesity may alter the function of innate and adaptive immune cells and weaken the host antiviral immune response to fight viral infections (see **Figure 2**).

### The influence of obesity on the generation of protective immune responses after vaccination

To prevent severe viral diseases, it is best to develop vaccines against them, followed by comprehensive vaccination. Currently, several arbovirus vaccines are available, and some are under development (150–155). The use of vaccines has major implications for the control of these diseases in arbovirus-endemic areas. The live-attenuated vaccine against yellow fever, 17D, is one of the most effective vaccines ever produced. It was found that children with severe protein deficiency had a significantly lower seroconversion rate for 17D (12.5%) than healthy children (83.3%) (156). To date, no study has observed the effects of obesity on the development of protective immune response to arboviral vaccines. However, several investigations report that obesity can decrease the induction of immune response to various viruses and toxins, including influenza, SARS-CoV-2, tick-borne encephalitis virus, hepatitis B virus, and tetanus toxins (147, 157–164).

In the past, obesity has been found to interfere with the induction of an effective protective immune response to influenza virus vaccines (158, 159, 165). When individuals with obesity were vaccinated with an inactivated trivalent influenza vaccine, lower antibody titers were observed in participants with obesity 12 months post vaccination (159). Cellular and humoral immune responses were well maintained in healthy individuals after vaccination, whereas lower influenza virus antibody titers and decreases in CD8+ T-cell activation were observed in individuals with obesity 12 months after vaccination (159). Garner-Spitzer et al. studied the immune response to tick-borne encephalitis (TBE) virus vaccine in obese and healthy-weight and individuals with obesity (164). They observed that adults with obesity had a greater initial increase in TBE-specific antibody titers at day 7 to day 28, followed by a sharp decline 6 months post TBE vaccination that correlated with high BMI and leptin levels. Recently, a non-peer reviewed study from Italy analyzed antibody titer production in a cohort of 248 healthcare workers (158 women, 90 men) after vaccination with the second dose of an mRNA vaccine against COVID-19 (BNT162b2, Pfizer) (163). The results of this study show that the humoral immune response was significantly stronger in individuals without obesity compared to participants who were classified as being overweight or obese (*p*<0.0001) (163). In another investigation, Watanabe et al., investigated the variables associated with serological response following COVID-19 mRNA vaccines and found obesity as one of the most important factors associated with lower antibody titers (166). These data highlight the impact of obesity on antibody titers, potentially impairing vaccine-conferred protective immune responses to viral vaccines. Based on these studies, individuals with obesity mount antigen-specific antibody responses equivalent to those of individuals of healthy weight at early time points post-vaccination, yet the antibody titers of individuals with obesity rapidly wane around a year post-vaccination. Thus, these insights suggest that altered vaccination schedules or higher vaccine formulation doses could benefit the durability of antibody responses primed in individuals with obesity. In addition, there is a possibility that people with obesity may be at higher risk for break through infections due to the impact of obesity on the priming of immune responses. Because of the continuous increase in arboviral infections worldwide and the rising obesity rates, future studies are needed to monitor the induction of the immune response after vaccination in people with obesity to determine the long-term protective role of arboviral vaccines. In addition, it is necessary to unravel the mechanism behind the poor immune response to vaccines and the development of severe disease in people with obesity to prevent severe cases and develop alternative strategies/therapies for people with obesity.

### Arbovirus infection in people with obesity

Obesity has been found to affect the immune response to viral infections such as influenza, SARS-CoV-2, and coxsackievirus (19, 167–171). Several studies have also reported the association between obesity and disease severity in arboviral infections (172). Padmakumar et al. analyzed 1,111 patients with confirmed CHIKV infection and found that disease was more severe in individuals with obesity and was associated with severe inflammatory sequelae (33). Comparative data from clinical trials revealed that CHIKV was associated with more severe polyarthralgia and took longer to improve in diabetic vs. non-diabetic patients (173, 174). In addition, we have infected healthy weight, obese, and malnourished mice with arthritogenic arboviruses from the genus *Alphavirus* (CHIKV, Mayaro virus and Ross River virus) and observed increased morbidity such as footpad swelling and weight loss in obese mice under all conditions (46). Furthermore, higher levels of viremia and RNAemia were seen in obese mice compared to controls 1 day post MAYV infection. However, the group of obese mice had lower levels of both infectious virus and viral RNA 3 days after MAYV infection compared to lean controls. A similar pattern was observed 3 days post CHIKV infection, where the group of obese mice had significantly lower RNAemia compared to lean controls (46). Thus, consistent with the epidemiological data in humans, individuals with obesity have worse disease outcomes during arthritogenic alphavirus infection and may also have altered viral replication kinetics.

Recently, Geerling et al. used an obese mouse model to investigate the influence of obesity on the development WNV disease (45). They found that the group of obese mice had a higher mortality rate and increased virus titers in the central nervous system compared to animals in the control group. In addition, they observed that obesity also deregulated the host acute adaptive immune responses, as obese female mice exhibited significant disruption of neutralizing antibody function (45). Thus, obesity may promote altered viral pathogenesis and decreased neutralizing capacity of antibodies.

Several studies also reported that patients with obesity with DENV infections were more likely to develop severe symptoms compared with DENV patients without obesity (34, 175, 176). Chuong et al. investigated the influence of nutritional status on DENV replication, immune protection, transmission, and disease severity in obese mice (44). They observed that severe DENV disease in obese mice was associated with high levels of proinflammatory cytokines. The obese mice had increased circulating levels of B-cell activating factor (BAFF), CCL5, CCL17, Chitinase-3-like 1, CXCL5, and IFN-α after DENV infection compared to animals in the control group. These cytokine imbalances might contribute to increase disease severity in patients with obesity by inducing vascular leakage and reduced platelet levels (177–180). Overall, these studies provide evidence that obesity significantly alters host immunity to arboviral infections, which likely contributes to increased disease severity.

**Figure 3:**
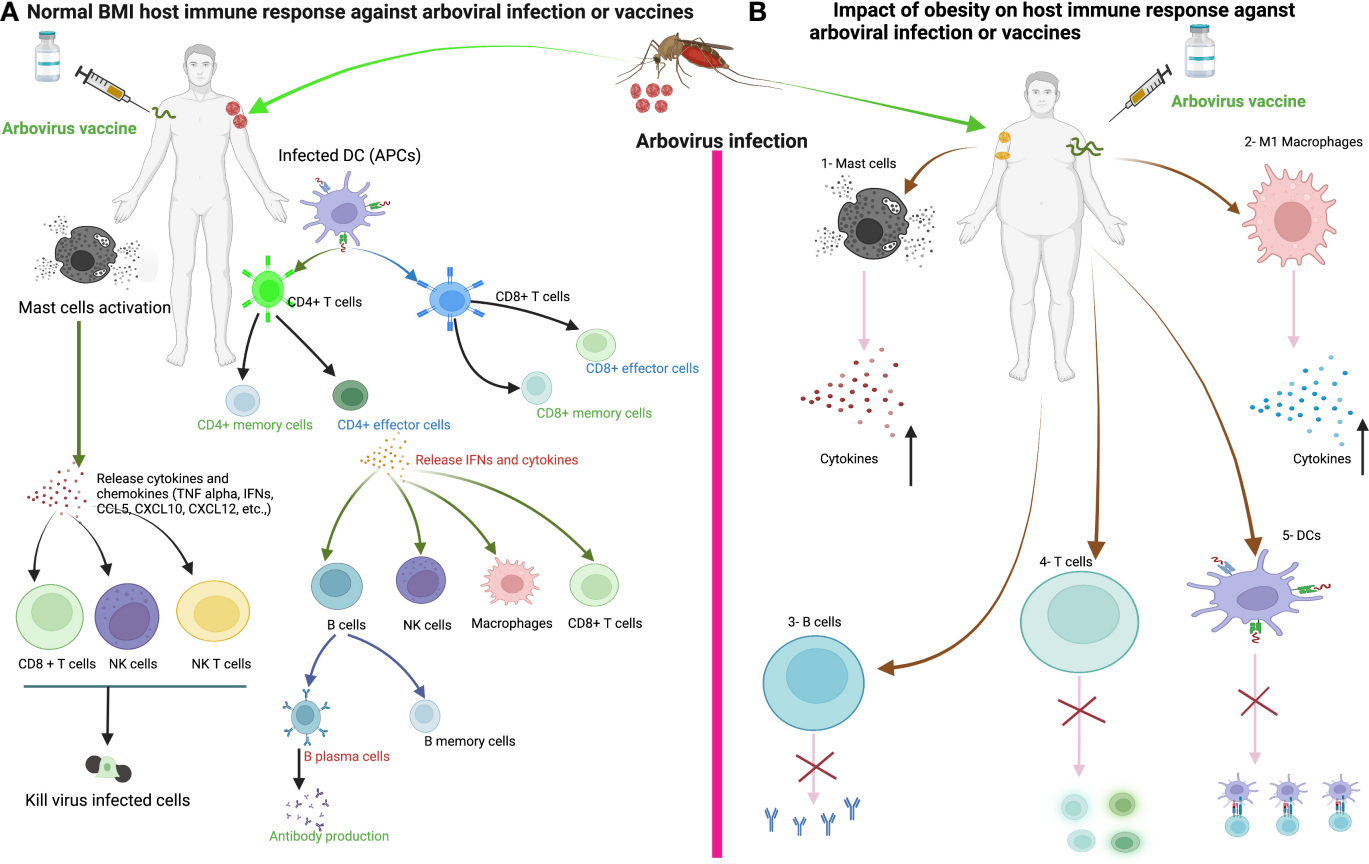
The immune response of healthy weight and obese individuals to arbovirus infection or vaccination. **(A)** The immune response of healthy individuals to arbovirus infection or vaccine. Sentinel cells of the immune system, such as dendritic cells, mast cells and macrophages, encounter the virus after infection from a mosquito bite or vaccine injection. Mast cells degranulate within minutes of recognizing the virus and release various cytokines/chemokines that activate other immune cells such as CD8+ T, natural killer (NK), NKT cells and macrophages against the viral infection to promote viral clearance. Dendritic cells or macrophages become infected and present viral antigens to CD4+ and CD8+ T cells to initiate an adaptive immune response. **(B)** We can speculate that the development of obesity disrupts the innate and adaptive immune cells during arboviral infection or vaccination, ultimately affecting the course of arboviral disease and the induction of the host immune response to the vaccine. 1-Increased production of proinflammatory cytokines from Mast cells in obesity. 2- Increase proinflammatory cytokines production from M1 macrophages in obesity. 3-Delayed antibody production. 4-Impaired activation of naïve T cells and Delayed CD4+ and CD8+ T cells response in obesity. 5-Impaired antigen presentation by DC in obesity.

In **Figure 3**, we illustrate the general immune response of the host to arbovirus infection. When the mosquito inoculates the virus into the human body, DCs and mast cells (MCs) in the epidermis encounter the virus. MCs degranulate and release cytokines (IFN-α and TNFα), chemokines (CCL5, CXCL10 and CXCL12) and proteases that play a critical role in the recruitment of CD8+ T, CD4+ T, NK and NKT cells to the site of infection (181, 182). The DCs, macrophages or monocytes are targets of arbovirus infection and act as antigen-presenting cells and release other cytokines, which in turn activate other innate and adaptive immune cells (183). DCs activated by arboviruses present antigens to CD4+ T and CD8+ T cells by upregulating co-stimulatory molecules such as CD80 and CD86 (184). Activated CD4+ T cells release IFN-γ, IL-4, IL-5, IL-10 or IL-12, which activate CD8+ T cells and B cells to clonally expand to produce CD8+ effector/memory and B plasma/memory cells (185). B plasma cells produce virus-specific neutralizing antibodies to prevent virus entry into the cells. Cytotoxic CD8+ T, NK and NKT cells, on the other hand, kill virus-infected cells and promote viral clearance. The development of obesity may disrupt the normal function of innate and adaptive immune cells during arbovirus infection or vaccination. We can speculate that the disruption of innate and adaptive immune cell functions during arbovirus infection or vaccination will ultimately affect the course of arbovirus disease and the induction of protective immune responses following infection or vaccination, which requires further investigation. This highlights the need for vigilance in the preventive or clinical management of viral infections in patients with obesity.

### Future perspectives

Obesity is a key public health problem. Nearly 13% of adults worldwide are obese and 40% are overweight. Obesity-induced chronic inflammation disrupts innate and adaptive immune cell functions, leading to impaired IFN and cytokine production, likely increasing the severity of viral disease. Recent epidemiological and experimental data strongly suggest that obesity is associated with increased disease severity in viral infections such as influenza virus, SARS-CoV-2, DENV, WNV, and CHIKV. The number of cases of arbovirus infections is steadily increasing worldwide. There are limited data on the host antiviral immune response and viral dynamics following arbovirus infection in individuals with obesity, highlighting the need for further studies to elucidate the cellular and molecular aspects of immunity that are compromised in obesity during arboviral infection and how these factors contribute to worsened disease outcomes. In addition, several studies have reported that individuals with obesity produce a poor protective immune response to vaccines or natural infections. Therefore, it is necessary to study the impact of obesity on antigen-specific immunity to ensure this population is protected following vaccination or natural infection. Obesity affects hundreds of millions of people around the world and a better understanding of the associated disorders will help scientists and physicians to develop specific therapeutic/prophylactic interventions to prevent immunopathology and disease progression in at-risk populations.

## Author contributions

All authors contributed to the article and approved the submitted version.

## Conflict of interest

The authors declare that the research was conducted in the absence of any commercial or financial relationships that could be construed as a potential conflict of interest.

## Publisher’s note

All claims expressed in this article are solely those of the authors and do not necessarily represent those of their affiliated organizations, or those of the publisher, the editors and the reviewers. Any product that may be evaluated in this article, or claim that may be made by its manufacturer, is not guaranteed or endorsed by the publisher.
